# Editorial: HBOC and high-risk screening: up-to-date

**DOI:** 10.1007/s12282-021-01284-3

**Published:** 2021-08-23

**Authors:** Masako Kataoka

**Affiliations:** Department of Diagnostic Imaging and Nuclear Medicine, Kyoto University Graduate School of Medicine, 54 Shogoin-kawaharacho, Sakyo-ku, Kyoto, 606-8507 Japan

Research on breast cancer risk revealed that 5–10% of breast cancer patients are genetically predisposed to cancers [1]. More than 25 years have passed since *BRCA1* was identified and cloned [2, 3]. *BRCA 1 and BRCA 2* are well-known susceptibility genes for breast cancer, which are also associated with ovarian and pancreatic cancers. Recent advancement in the mechanism of hereditary breast cancer has changed the clinical approach to patients and families who are considered “high risk” for breast cancer. Genetic testing is becoming a feasible option to accurately estimate their own risk and to optimize screening/surveillance strategy.

Due to the accumulating evidence and continuous efforts by professional societies, several medical examinations, and treatments related to HBOC have become covered by insurance in Japan [4]. These include BRCA genetic testing to determine eligibility for PARP inhibitor therapy and for patients diagnosed with breast or ovarian cancers who are likely to have a genetic predisposition. For patients diagnosed as HBOC risk-reduced mastectomy (RRM), breast reconstruction, and risk-reduced oophorectomy (RRSO) are covered by insurance. MRI-guided biopsy was also covered by insurance.

Nowadays, clinicians and professionals who are involved in the management of breast cancer need to have sufficient knowledge of hereditary breast and ovarian cancer (HBOC) and other high-risk conditions of breast cancer to support patients’ choice. The benefit and harm of breast cancer screening/surveillance vary depending on individual risk of breast cancer. Conducting genetic testing and knowing the results affect patients’ life and families’ life and therefore fully-informed decisions should be made.

As a journal dedicated to breast cancer, we have dealt with the issues related to the high risk of breast cancer [5–10]. This special feature aims to cover important knowledge about HBOC that may be necessary in the clinic. Yoshida covers key factors for breast cancer genetics including BRCA 1 BRCA2 and other less-common risk genes [1]. The paper mentions the difference between western countries and Asian countries in frequencies of breast cancer-related genes, including higher prevalence of BRCA2 gene among Asian countries [11]. Since breast cancers associated with BRCA1 and 2 have different clinico-pathological features, their management may need to be adjusted for each group. Knowing other genes predisposing to cancer may be crucial for preventing future cancer in the breast and other organs. Genetic information varies depending on ethnicity, and we need to know the frequency of diseases and genes that are unique to us.

Screening strategy by imaging is a crucial component of HBOC management. The paper by Tozaki and Nakamura describes the current status of breast cancer screening in high-risk women in Japan [4]. Based on their prospective research project, they reported the data of image-based screening among Japanese women and the value of MRI surveillance. In addition, they claimed that different surveillance strategies should be used for BRCA 1 and 2 mutation carriers considering their different pattern of developing cancer with different imaging features. Triple-negative cancer is more common among BRCA1 mutation carriers and can appear benign like fibroademona, while the luminal-type breast cancer is more common in BRCA2 mutation carriers presenting calcification on mammogram in half of cases.

More practical side of MRI-guided vacuum-assisted biopsy (VAB) is covered in this featured paper [12]. There are a limited number of facilities which can regularly perform MRI-guided biopsy. However, MRI-guided VAB is the only method for MRI-only detected lesions often found on high-risk screening. In addition to the practical aspect, the data on MRI-guided- biopsy are nicely summarized.

Worldwide, various professional societies in many countries issue guidelines for breast cancer screening for normal and elevated risk of breast cancer. Onishi et al. encompass the guidelines and compare these guidelines [13]. There are variations in criteria among various groups/ country. These are useful guides for us to establish screening guidelines in our country.

I hope that these special feature papers are useful for reviewing up-to-date knowledge of HBOC for readers who are interested in this topic and are helpful in supporting those with high -risk of breast cancer.
